# Muscle Fibers, Free Amino Acids, and Enhanced Mitochondrial Function Explain the Unique Meat Quality of Tibetan Pigs

**DOI:** 10.3390/foods14213591

**Published:** 2025-10-22

**Authors:** Hao Li, Jie Wu, Yizhi Luo, Zekai Yao, Xinxin Li, Yebiao Ji, Baohong Li, Haiyun Xin, Bin Hu, Sutian Wang, Leiyan Cheng, Ying Wang, Ming Yang, Zhenfang Wu, Jie Yang, Enqin Zheng, Fanming Meng

**Affiliations:** 1 State Key Laboratory of Livestock and Poultry Breeding, Guangdong Provincial Key Laboratory of Animal Breeding and Nutrition, Institute of Animal Science, Guangdong Academy of Agricultural Sciences, Guangzhou 510640, China; haolee97@hotmail.com (H.L.); wujiezi163@163.com (J.W.); 14770011284@163.com (Z.Y.); lixx220014@163.com (X.L.); jeremiah824@163.com (Y.J.); boohom@163.com (B.L.); xinhaiyun503@163.com (H.X.); 14749392070@163.com (B.H.); wstlyt@126.com (S.W.); cleiyan@163.com (L.C.); 2 College of Animal Science and National Engineering Research Center for Breeding Swine Industry, South China Agricultural University, Guangzhou 510642, China; wzfemail@163.com (Z.W.); jieyang2012@hotmail.com (J.Y.); 3 Institute of Facility Agriculture, Guangdong Academy of Agricultural Sciences, Guangzhou 510640, China; luoyizhi@gdaas.cn; 4 Guangzhou Customs Technology Center, Guangzhou 510623, China; s1610587816@163.com; 5 College of Animal Science and Technology, Zhongkai University of Agriculture and Engineering, Guangzhou 510550, China; yangming@zhku.edu.cn

**Keywords:** Tibetan pig, Duroc pigs, free amino acids, histomorphology, proteomics

## Abstract

The mechanistic underlying the favorable meat quality of Tibetan pigs has not been fully elucidated. This study integrated flavor chemistry, histomorphology, and proteomics to explore the structural and molecular features of their meat. Longissimus dorsi samples from Tibetan and Duroc pigs (*n* = 6 each biological replicates) were quantitatively analyzed for amino acid profiling, histological assessment, and proteomic characteristic. Statistical approaches included weighted correlation network analysis, *t*-tests, and functional enrichment. Tibetan pork contained 34 mg/100g more total free amino acids, notably sweet-tasting Ala (+49.2%) and Thr (+32.2%). Muscle fiber density was >250% higher and diameter > 30% smaller, indicating finer texture. Proteomics revealed 149 upregulated proteins, including 57 mitochondrial differentially expressed proteins (DEPs)—11 of which belonged to electron transport chain complexes (e.g., NDUFAB1, COX2). The significant enrichment of oxidative phosphorylation pathways may be associated with mitochondrial efficient energy metabolism under hypoxic in Tibetan pigs, potentially linking to the breed’s unique meat characteristics. Ala levels showed strong correlations with metabolic and structural protein modules. The finer fibers and mitochondrial protein profile of Tibetan pigs contribute to higher amino acid content and meat quality. This structural–metabolic–flavor axis supports both hypoxia adaptation and high meat quality. Given the central role of mitochondrial electron transport chain (ETC) proteins in energy metabolism and Ala in flavor presentation, their synergistic action provides a molecular bridge between hypoxia adaptation and meat quality. Therefore, this study suggests that ETC and Ala may serve as key biomarkers for meat quality differences, offering new perspectives for meat quality research.

## 1. Introduction

Pork is an important source of dietary protein worldwide, and consumers are increasingly concerned about the quality of meat. Improving muscle quality remains a key scientific goal [1,2,3]. Pork quality depends on many factors including pig genotype, nutrition, enclosure and slaughter conditions. Meat maturation and storage also influence quality [2]. The distinct meat characteristics of Duroc pigs, prized for their intramuscular fat and growth efficiency, and Tibetan pigs, valued for their cold tolerance and firm, flavorful meat, make them ideal models for uncovering the molecular basis of divergent meat quality. The mentioned benefits of Tibetan pigs (more total free amino acids, finer texture, and others) in comparison with Duroc pigs are not compensated for by different rearing methods until 10 months of age, and Duroc pigs until 6 months of age. A study compared the significant differences in meat quality between Tibetan pigs and Duroc pigs through proteomics, investigating breed-specific metabolic and structural characteristics [3,4,5]. Amino acids significantly influence the taste of pork. Amino acid levels that better meet human nutritional requirements enhance meat’s health benefits and immunomodulatory properties. Certain amino acids such as Lysine, Alanine, and Threonine make important contributions to the meat’s sweetness [6]. A study identified 5 cytoskeletal proteins, 3 protein-binding proteins, and 7 metabolic enzymes as potential biomarkers of lamb tenderness by determining the proteome using bioinformatic co-analysis, this indicates that proteomics can serve as a screening method for animal tenderness markers, and the same applies to pigs [7]. Recent transcriptome mapping identified key genes contributing to porcine skeletal muscle differentiation [8]. However, there are few multi-omics studies linking the amino acid profiles, proteomes, structure, and flavor of Tibetan pigs and Duroc pigs. This study was initiated based on the differences in the content of flavor-presenting amino acids in the longissimus dorsi muscle between Tibetan and Duroc pigs. We employed expression profiling and TMT-based proteomics to analyze the differences in gene expression and protein abundance in this tissue. Subsequently, bioinformatics approaches were integrated to screen for candidate genes influencing muscle differentiation and flavor traits between the two breeds. This study aims to elucidate molecular mechanisms underlying meat quality differences between Tibetan and Duroc pigs, providing new insights for meat science research.

## 2. Materials and Methods

### 2.1. Laboratory Animal

The experimental animals selected for this study were six Duroc pigs and six Tibetan pigs each, which were introduced from Gansu Province, China, to the Institute of Animal Science, Guangdong Academy of Agricultural Sciences, China, for breeding in 2018. Both Duroc pigs and Tibetan pigs were raised in 2020 within the Institute of Animal Science, Guangdong Academy of Agricultural Sciences, China. The feeding style and nutritional standards were the same, all were reared in a semi-open system, and the diets were purchased from Guangdong Chia Tai Conti Co., Ltd. (Guangdong, China), with raw material composition of maize, wheat sub-flour, soybean meal, soybean oil, amino acid salts, enzymes and minerals, etc., and the nutritional standards were crude protein ≥ 17%, crude fiber ≥ 8%, crude ash ≤ 8%, and lysine content ≥ 0.9%. To meet market demand and preserve the meat at its peak quality, each breed is slaughtered during its optimal fattening stage [9,10,11]. Tibetan pigs were reared until 10 months of age, and Duroc pigs were reared until 6 months of age, with fasting the day before slaughter, but with free access to water [12,13].

### 2.2. Sample Collection

Samples were collected as previously described by Li et al. [14], in brief, the longissimus dorsi muscle sample for histochemical analysis was dissected from the tenth thoracic vertebra on the right-side carcass within half an hour postmortem. Tissues were sectioned using a cryostat microtome (RM2235 cwEU, Leica Microsystems Shanghai Co., Ltd., Shanghai, China). The muscle sample was placed on a piece of A4 PVC binding cover, and a 2  ×  0.5  ×  0.5 cm^3^ sample was taken along the muscle fiber direction, then immediately fixed in 10% buffered neutral formalin solution for 24 h. After fixation of the specimens, they were dehydrated in alcohol, cleared in xylene, infiltrated and finally embedded in paraffin. The sections were cut at 3 μm thickness and stained with hematoxylin and eosin (H&E) for general histological study. An additional 100 g of longissimus dorsi was collected in a plasticized bag for free amino acid assay. Muscle tissue samples (1 g each) were collected into cryovials, immediately snap-frozen in liquid nitrogen, and subsequently stored at −80 °C for proteomic analysis. Six pigs per breed, with three replicate samples collected from each pig for combined analysis.

### 2.3. Free Amino Acid Measurement

Free amino acid (FAA) determination was carried out using ninhydrin post-column derivatization ion exchange chromatography for the determination of FAA in the longissimus dorsi of the back, weighing the appropriate amount of sample and adding 3 mL of sulfosalicylic acid (20%, *w*/*v*) homogenate low-temperature centrifugation for 15 min (4 °C, 12,000× *g*), filtering the supernatant using 0.22 μm aqueous filtration membrane, and adding the standard working solution to the amino acid autoanalyzer, and calculating the free amino acid concentration according to the instructions of the instrument.

### 2.4. Muscle Fiber Number, Diameter, and Area Statistics

Five photographs of different cross-sections from each muscle were taken. The samples were determined by using Image-J software (v13.0). The mean number of fibers per area was obtained by counting the total number of fibers (TNF) in five areas per sample. The mean of approximately 200 fibers in five random fields for each muscle was measured to estimate the fiber diameters (μm) and fiber areas (μm^2^).

### 2.5. Protein Extraction and Data Quality Control

The protein extraction method was referred to the previous method [15]. The standard curve was plotted using the absorbance of the standard protein solution and the protein concentration of the samples was calculated. Using the Bradford protein quantification kit, BSA standard protein solution was prepared with a concentration gradient ranging from 0–0.5 µg/µL. 20 µg of each protein sample to be tested was subjected to 12% SDS-PAGE gel electrophoresis, and the conditions of electrophoresis of the separating gels were 120 V for 90 min. At the end of electrophoresis, the samples were subjected to Coomassie Brilliant Blue staining, destained until the bands were clear, and followed by proteolytic digestion and liquid-quality detection [16]. All resulting spectra were searched in the Uniprot protein database using Proteome Discoverer 2.2 (PD2.2, Thermo, Waltham, MA, USA). The search library parameters were set to a mass tolerance of 10 ppm for precursor ions and 0.02 Da for fragment ions. Peptide Spectrum Matches (PSMs) with a confidence level of 99% or more and proteins containing at least one unique peptide were retained, and peptides and proteins with an FDR > 1% were removed.

Protein quantification results were statistically analyzed using the *t*-test, and proteins with significant quantitative differences between Tibetan pigs and Duroc pigs (*p* < 0.05, FC ≥ 1.5) were defined as differentially expressed proteins (DEPs). The identified proteins were enriched and analyzed using CO and KEGG, while the subcellular localization of DEPs was performed on the Cell-PLOC 2.0 website [17]. DEPs were represented using volcano plots and clustered according to grouping [18]. Finally, the DEPs were annotated to the STRING database to construct a Protein–Protein Interaction Networks (PPI), and the confidence score was set to high confidence (confidence > 0.700).

### 2.6. Weighted Gene Co-Expression Network Analysis (WGCNA)

Weighted correlation network analysis (WGCNA) can be utilized to identify clusters (modules) of highly associated genes, summarize such clusters based on the module eigengene, and relate modules to external clinical traits [19]. In brief, we preprocessed the raw gene expression profile data to remove low-quality reads, and correct for batch effects and technical variations between samples by quantifying expression levels, and normalizing the data. Then, a correlation or similarity matrix is constructed to represent gene relationships. Using a soft thresholding approach, highly co-expressed gene modules are identified. The modules are analyzed for preservation and significance. Hub genes with high connectivity within each module are identified. Functional enrichment analysis is performed to understand the biological processes associated with the modules and hub genes. Correlation networks can be used to screen candidate hub genes [20].

### 2.7. Statistical Analysis

Data are presented as least squares means with standard errors for growth performance and meat quality traits analyzed using a general linear model (GLM). For proteomic and amino acid data, differences between breeds were assessed using a paired *t*-test of SAS (v26, Cary, NC, USA). The paired design was implemented to control for batch effects, as animals from both breeds were processed concurrently under identical conditions. The assumptions of normality and homogeneity of variances for all *t*-tests were verified using Shapiro–Wilk and Levene’s tests, respectively. Values of *p* < 0.05 were considered to indicate statistically significant differences. The results are presented as least squares means with standard errors.

## 3. Results

### 3.1. Descriptive Results Statistics of Free Amino Acids

The content of FAA and the main flavor presenting characteristics in the longest dorsal muscle in Duroc pigs and Tibetan pigs were shown in Table 1. The data were analyzed by one-way ANOVA through the GLM procedure of SAS software. The *p*-value of the one-way analytical test for FAA in Duroc pigs and Tibetan pigs was 2.66 × 10^−2^, and the *p*-value of the one-way test for each FAA was 2.15 × 10^−7^, and the Pearson correlation coefficient of the two groups of samples was 0.96. In this study, the total FAA content of Tibetan pigs was 200.65 mg/100 g, and the total FAA content of Duroc pigs was 166.28 mg/100 g (↓20.67%). The most abundant FAAs in the longest dorsal muscle of Tibetan pigs were alanine (Ala) and threonine (Thr), both with sweet flavor, 37.42 mg/100 g and 30.20 mg/100 g, respectively, which were twice as much as and more than the other FAA. This heightened sweetness in Tibetan pig enhances the overall flavor complexity and palatability, providing a mellower and more harmonious taste experience compared to Duroc pork. Sweet FAA was the taste sensation with the highest percentage of FAA in the longest dorsal muscle of Tibetan pigs, accounting for 46.15% of the total FAA, followed by umami FAA with 20.75%. Similar to previous research findings, Ala and Thr also exhibited the highest FAA content in the longest back muscle of Duroc pigs, but their levels were 49.20% and 32.22% lower than those in Tibetan pigs, respectively. Comparative analysis revealed that the longissimus thoracis of Tibetan pigs surpassed Duroc pigs in total free amino acids (↑34 mg/100 g), sweet-tasting FAA (92.59 vs. 62.70 mg/100 g), and essential amino acids (↑20 mg/100 g, +25.97%), while both breeds showed a similarly high proportion (>75%) of flavor-presenting FAA.

### 3.2. Muscle Fiber Number and Area Statistics

**Figure 1:**
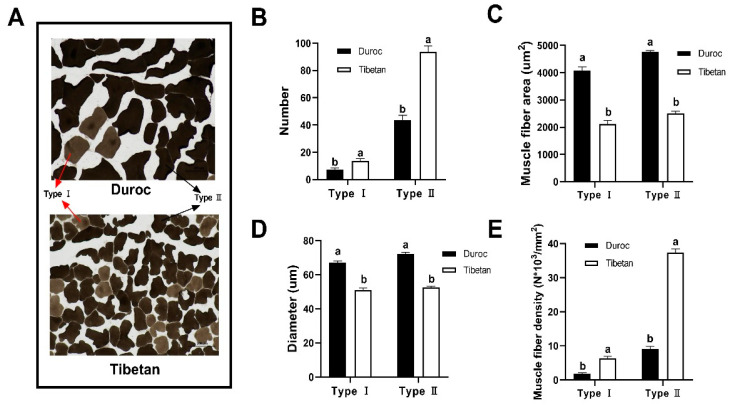
Muscle fiber counts determined using ImageJ v13.0. (**A**) 200X frozen section of the longest muscle of the longissimus dorsi. Number (**B**), area (**C**), diameter (**D**)**,** and density (**E**) of type I and type II muscle fibers. ^a,b^ Data at the same column with different lowercase letters indicated significant differences (*p* < 0.05).

The muscle fiber architecture of Tibetan and Duroc pigs was quantitatively assessed through manual morphometry (Figure 1A). As shown in Figure 1B, the number of type I (14/7) and type II (94/44) muscle fibers was significantly greater in Tibetan pigs than in Duroc, while the area (Figure 1C) and diameter (Figure 1D) of muscle fibers were significantly greater in Duroc than in Tibetan pigs. These structural differences reflect a distinct muscle phenotype: Tibetan pigs possess finer and more densely packed fibers, as evidenced by lower mean fiber areas (type I: 2123.57 vs. 4075.89 μm^2^; type II: 2508.43 vs. 4757.41 μm^2^) and diameters compared to Duroc pigs. This morphological profile resulted in markedly higher myofiber density in Tibetan pigs, with type I and type II densities reaching 258.43% and 303.31% of those in Duroc, respectively (Figure 1E). The finer texture and higher density of muscle fibers are key structural determinants of meat tenderness, as they reduce the required shear force during mastication. Moreover, the elevated proportion of oxidative type I fibers in Tibetan pigs aligns with their enhanced mitochondrial metabolic capacity, suggesting an adaptive muscle physiology that supports sustained activity under hypoxic conditions while also contributing to favorable post-mortem meat quality traits such as color stability.

### 3.3. Results of Proteomic Analysis

**Figure 2:**
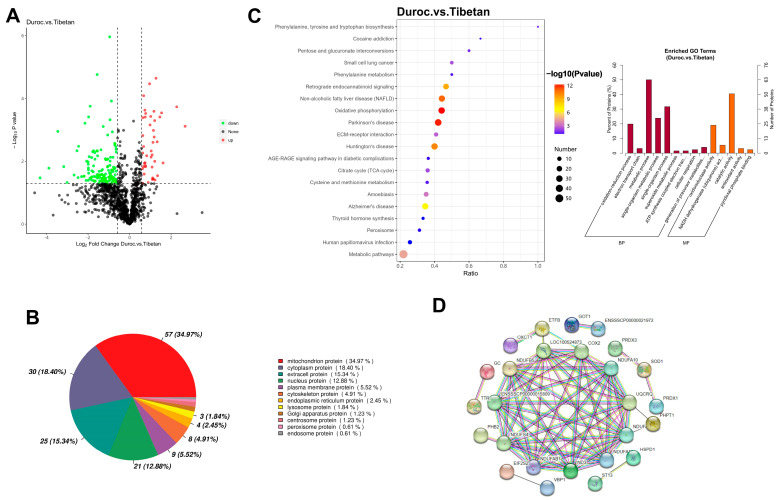
Proteomic analysis. (**A**) Volcanic map of DEPs. (**B**) Subcellular localization analysis. (**C**) GO and KEGG enrichment analysis. (**D**) Protein Interaction Networks.

The initial 1,587,449 secondary spectra were filtered to yield 416,529 validated spectra containing 20,335 peptides, resulting in the identification of 1878 proteins. All samples were above 1100 proteins, and the average protein number was found to be 1157 in Duroc pigs and 1218 in Tibetan pigs. A total of 193 DEPs were identified in this study, and 44 proteins were highly expressed in Duroc pigs, and 149 proteins were highly expressed in Tibetan pigs (Figure 2A). Proteins involved in metabolic processes may regulate the abundance of flavor precursors, such as free amino acids and nucleotides, directly affecting taste perception. The results of subcellular localization showed that of the 193 DEPs between species, 163 had annotated information in the Cell-mPLOC 2.0 website, of which 57 proteins were mitochondrial proteins (Figure 2B). GO was predominantly enriched in metabolic processes and catalytically active pathways, and KEGG pathway analysis indicated that DEPs were predominantly enriched in the oxidative phosphorylation pathway (Figure 2C). PPI analysis displayed 26 DEPs with interactions (*p* < 1.0 × 10^−16^), of which 23 protein structures were known with an average clustering coefficient of 0.448 (Figure 2D). A total of 11 proteins, including NDUFAB1, NDUFA10, LOC100524873, COX2, ND3, NDUFS4, NDUFA12, ENSSSCP000015809, UQCRQ, NDUFA8, and NDUFB5, were at the core nodes of the interaction network, and the significant proteins and their expressions are shown in Table S1. The upregulation of 11 ETC core proteins (e.g., NDUFAB1, COX2) enhances mitochondrial respiratory efficiency, which is crucial for sustaining aerobic metabolism in muscle.

### 3.4. Integration of Free Amino Acid Profiles and Proteomic

**Figure 3:**
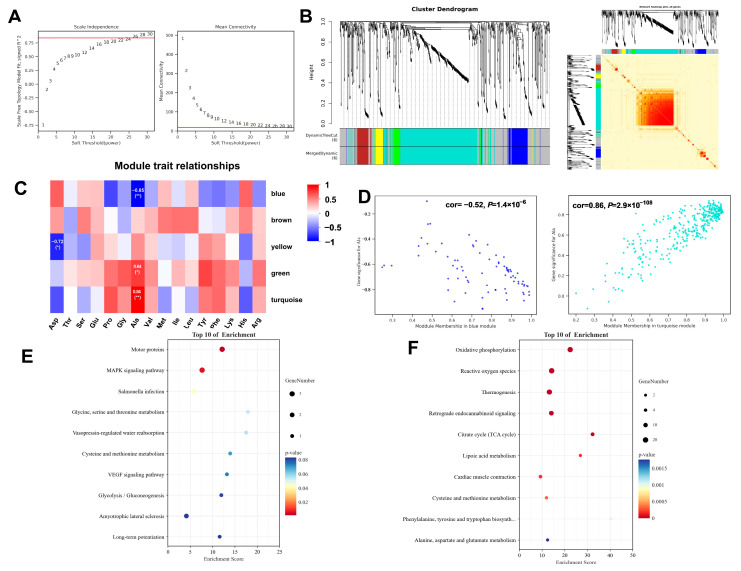
Weighted gene co-expression network analysis. (**A**) Determination of the optimal soft threshold utilized for gene clustering, red line represents the soft threshold. (**B**) Dendrogram of all differentially expressed genes from the dynamic tree cut. (**C**) Analysis of the relationships between modules and traits. (**D**) Dot plots illustrating the distribution of genes within the green and turquoise modules. (**E**) KEGG enrichment analysis of the key genes in blue module. (**F**) KEGG enrichment analysis of the key genes in turquoise module.

To elucidate protein modules associated with flavor-related amino acids, a weighted gene co-expression network analysis (WGCNA) was performed using a dataset comprising 10 samples. This method groups highly correlated proteins into “modules” (clusters), each represented by a synthetic profile called the “module eigengene. A soft-thresholding power was applied to transform the correlation matrix into an adjacency matrix, which emphasizes strong correlations while penalizing weak ones, thereby constructing a biologically more relevant co-expression network. Based on the standard of a scale-free network, a value of 26 was selected as the soft threshold, and 0.84 as the independence, which ensured the biological relevance of the co-expression network (Figure 3A). Subsequently, a correlation matrix was constructed, and genes were grouped into six distinct modules (Figure 3B). With amino acids as phenotypic information, the Pearson correlation coefficient was calculated between the module and phenotypic information (Figure 3C). Notably, alanine (Ala), an amino acid known to contribute significantly to sweet taste perception, exhibited a strong negative correlation with the blue module (r = −0.85, *p* < 0.01) and a strong positive correlation with the turquoise module (r = 0.84, *p* < 0.01) (Figure 3D). KEGG pathway enrichment analysis was conducted for the blue and turquoise modules overlapping with the DEPs. The blue module was significantly enriched in Motor proteins and the MAPK signaling pathway (Figure 3E), while the turquoise module showed predominant enrichment in Oxidative phosphorylation, Reactive oxygen species, Thermogenesis, and the TCA cycle (Figure 3F). The identification of these protein modules provides vital insights into the meat quality and amino flavor.

## 4. Discussion

The growing consumer demand for high-quality pork has intensified the focus on its sensory attributes, particularly flavor. In this context, FAAs play a pivotal role, as their composition and concentration are key determinants of taste. Different FAAs act synergistically to create the overall flavor profile, while their collective content also serves as a significant indicator of the meat’s nutritional value [21]. The total FAA and amino acid content of sweet flavors were higher in local pig breeds than in lean pigs, which is in line with the conclusion of previous study. Therefore, the superior meat quality of Tibetan pigs is evidenced not only by a favorable FAA profile that promises a rich, sweet-umami taste experience but also by a robust EAA content that underscores its high nutritional worth [22,23]. Tibetan pork is contained higher of essential amino acids, and its nutritional value is greater than that of other breeds of pork. In this study, the Tibetan pig pork dorsal longest muscle had the highest amount of Ala and Thr, both of which showed sweetness in taste. The high level of Ala plays a dual role. As a sweet-tasting amino acid, it directly enhances the sweet flavor notes in the meat, improving its taste. Additionally, Ala is a key molecule in energy metabolism, and its abundance serves as an indicator of active metabolic processes in the muscle, which are linked to the meat’s overall quality [24]. Previous studies have reported that appropriate dietary threonine supplementation can improve muscle fiber characteristics and hardness. This finding provides a theoretical basis for enhancing meat quality through nutritional regulation. Consequently, supplementing feeds with FAAs or their derivatives emerges as a viable strategy not only to improve flavor and meat quality but also to confer antioxidant and stress-reducing benefits [23]. Amino acids play multifunctional roles in shaping pork quality. As flavor precursors, they generate characteristic tastes through thermal degradation and the Maillard reaction. In terms of texture, they influence muscle physiology and post-mortem proteolysis, affecting tenderness and juiciness. Their antioxidant capacity helps mitigate oxidative rancidity, preserving sensory quality. From a health perspective, they elevate the meat’s nutritional index by enriching its essential amino acid content, making dietary supplementation a powerful tool for comprehensive quality improvement [23,25]. The total content of arginine, leucine and proline in Tibetan pigs in this study was about 5 mg/100 g higher than that of Duroc pigs (↑15.92), these amino acids improve protein deposition and meat texture [26], enhance antioxidant [27,28], and are beneficial to human health [29].

### 4.1. Muscle Fiber Microstructure and Its Implications for Meat Texture

The content of free amino acids—critical flavor precursors—is linked to myofiber type, with a high proportion of red myofibers often indicating greater abundance. Simultaneously, myofiber number and type jointly determine key quality attributes: more fibers enhance tenderness, while the type affects metabolism and water retention, thereby influencing juiciness and flavor potential [30]. In this study, we found that the morphology of muscle fibers in the longest dorsal muscle of Tibetan pigs was significantly different from that of Duroc pigs. Tibetan pigs exhibited a finer and firmer myofibrillar structural, with significantly smaller area and diameter of both type I and II myofibers than Duroc pigs. This microstructural characteristic is physiologically significant for meat quality. A smaller individual fiber diameter is typically associated with a denser spatial arrangement. This finding aligns with previous studies [31,32], which suggest that a greater density of finer fibers increases the overall surface area of the myofiber-connective tissue interface, a key microstructural factor that mechanically contributes to enhanced meat tenderness.

### 4.2. Breed-Specific Muscle Development

A pivotal finding was the significantly higher total number of muscle fibers per unit cross-sectional area in Tibetan pigs. Notably, the counts of both type I and type II fibers showed a synchronous increase. This phenomenon indicates the occurrence of muscle fiber hyperplasia during development in Tibetan pigs. This provides cellular-level evidence supporting the notion that Tibetan pigs may activate a stronger or prolonged myoblast proliferation pathway compared to breeds like Duroc, which typically exhibit muscle growth primarily through fiber hypertrophy (an increase in fiber size) [33].

### 4.3. Linking Structure to Sensory Quality

The differences in muscle fiber properties uncovered in this study provide a coherent microscopic explanation for the superior texture of Tibetan pork. The combination of smaller fiber diameters and higher fiber density is widely correlated with a finer and more delicate meat texture in culinary science. We therefore conclude that this specific structural architecture—comprising finer, more numerous fibers—forms the fundamental structural basis for the superior tenderness and, consequently, the enhanced overall eating quality consistently associated with Tibetan pig muscle.

The proteomic profile of Tibetan pigs revealed a more complex regulatory network, with 1218 proteins identified on average—significantly more than the 1157 in Duroc pigs. Among the 193 DEPs, 77.2% (149) were upregulated in Tibetan pigs, indicating breed-specific molecular regulation. Notably, 34.97% (57/163) of localized DEPs were mitochondrial proteins, a finding consistent with the enrichment of oxidative phosphorylation in KEGG analysis and catalytic/metabolic processes in GO terms. These multi-level results collectively indicate a pronounced reprogramming of mitochondrial energy metabolism in Tibetan pigs. Further protein–protein interaction (PPI) analysis identified 11 hub proteins—all components of the mitochondrial electron transport chain (ETC), including NDUFAB1, COX2, and ND3. The high interaction significance (*p* < 1.0 × 10^−16^) and network clustering coefficient (0.448) suggest strong co-expression and functional synergy among ETC complexes [34]. Previous study found that Tibetan pig and Duroc skeletal muscle differential proteins were mainly enriched during locomotor activity, myosin regulation, by ubiquitination 4D proteomics, similar to the findings of the present study [35]. The proteomics directly echoed the myofiber morphology: the fine, dense muscle fibers of Tibetan pigs demand a denser mitochondrial distribution and stronger oxidative phosphorylation capacity for metabolic support. The synergistic up-regulation of mitochondrial ETC components points to an adaptive metabolic strategy that supports muscle contraction in hypoxia, may offer a plausible mechanism for the breed’s enhanced exercise endurance.

The protein networks identified in this study elucidate the molecular basis for the superior meat quality of Tibetan pigs. We found that Ala, a key sweet-tasting compound in meat, is closely linked to two functional protein groups [36,37]. One group, enriched in structural and motor proteins, reflects the distinct muscle architecture of Tibetan pigs. These proteins are associated with muscle fiber type and stress adaptation, which may influence both muscle development and the release of flavor precursors such as Ala during meat processing [38]. The other protein group is primarily involved in mitochondrial energy metabolism, including oxidative phosphorylation and the TCA cycle. This indicates more active and efficient energy production in Tibetan pigs, which helps maintain muscle vitality and delays acidification after slaughter. Such metabolic characteristics contribute to better water retention, steadier pH decline, and improved texture and flavor stability in the final meat product [39]. Together, these findings reveal how Tibetan pigs’ genetic background shapes both their muscle structure and metabolic traits, which collectively enhance eating quality through higher sweet amino acid accumulation and optimized post-mortem metabolism [40].

## 5. Conclusions

In conclusion, this integrated study provides multi-level insights into the formation mechanism of the superior meat quality in Tibetan pigs by combining muscle histology, proteomics, and flavor chemistry. The results indicate that the enhanced taste and nutritional value of Tibetan pig are closely associated with its significantly higher content of free amino acids (especially Ala and Thr) compared to Duroc pig. Histologically, the finer texture of Tibetan pig is supported by a denser and smaller muscle fiber structure. At the molecular level, the upregulation of mitochondrial proteins and activation of the oxidative phosphorylation pathway appear to facilitate efficient energy metabolism under hypoxic conditions, which may contribute to the observed meat traits. Furthermore, WGCNA revealed that Ala accumulation is correlated with modules related to oxidative metabolism and structural proteins. These omics analyses provide potential molecular targets for understanding fleshy tissue formation. However, this study has several limitations that need to be addressed. For instance, the sample size was relatively small, the breeds and ages of the pigs varied, and the analysis focused solely on the longissimus dorsi muscle. Therefore, future research will focus on increasing sample sizes, selecting pig breeds with similar fattening periods, and expanding protein-related functional tests. Efforts will be made to link other tissues with sensory evaluations, which may inform genetic selection and nutritional strategies aimed at improving meat quality in pork production.

## Figures and Tables

**Table 1:** Determination of free amino acids in Tibetan and Duroc pigs.

Amino Acid Type	Duroc Pigs	Tibetan Pigs	Flavor Characteristics
Phe	14.55 ± 1.96	17.31 ± 5.84	umami
Tyr	9.25 ± 2.56	11.25 ± 3.10	umami
Glu	15.43 ± 3.07	13.08 ± 3.31	Umami, acidity
Asp	1.28 ± 0.57	0.72 ± 0.86	acidity
Gly	9.31 ± 1.12	10.04 ± 0.35	sweetness
Ala	25.08 ± 1.31 ^b^	37.42 ± 2.57 ^a^	sweetness
Ser	8.35 ± 0.98	9.37 ± 0.66	sweetness
Thr	22.84 ± 3.50 ^b^	30.20 ± 3.01 ^a^	sweetness
Pro	3.38 ± 0.37 ^b^	5.56 ± 0.35 ^a^	Sweetness, bitterness
Leu	16.58 ± 2.75	16.22 ± 1.91	bitterness
Val	6.66 ± 0.29 ^b^	8.90 ± 1.37 ^a^	-
Met	7.24 ± 0.72	7.11 ± 0.46	-
Ile	5.45 ± 0.56	6.08 ± 0.61	-
Lys	8.34 ± 0.82 ^b^	12.25 ± 0.37 ^a^	-
His	3.62 ± 0.21	3.57 ± 0.85	-
Arg	8.81 ± 0.83 ^b^	11.57 ± 0.59 ^a^	-
total amino acids	166.28 ± 15.38 ^b^	200.65 ± 13.97 ^a^	

^a,b^ Data at the same column with different lowercase letters indicated significant differences (*p* < 0.05).

## Data Availability

Dataset available on request from the authors.

## Supplementary Materials

The following supporting information can be downloaded at: https://www.mdpi.com/article/10.3390/foods14213591/s1, Table S1: Important proteins and expression.
