# Supplementary material for: Muscle Fibers, Free Amino Acids, and Enhanced Mitochondrial Function Explain the Unique Meat Quality of Tibetan Pigs
Source: Foods. 2025 Oct 22;14(21):3591. doi: 10.3390/foods14213591 (PMC12610663; doi:10.3390/foods14213591)
Supplement: Supplementary file 1 [file foods-14-03591-s001.zip › foods-3918001-supplementary.pdf]

Supplementary

Table 1. Important proteins and expression.

Protein	Gene	Fold Change	*P*value	Expression[a]
F1SQP4	*NDUFA12*	0.234	0.040	down
A0A4X1UIS9	*PLCD4*	0.255	3.868×10^{-4}	down
A0A4X1VUZ7	*GPX4*	0.269	1.751×10^{-4}	down
A0A287AMN2	*NDUFS4*	0.297	0.022	down
F1S3W0	*LOC100524873*	0.319	0.042	down
F1SLR1	*NDUFA8*	0.344	0.032	down
F1SGC6	*NDUFB5*	0.369	0.042	down
A0ST13	*ND5*	0.393	0.020	down
F1SIS9	*NDUFA10*	0.407	0.042	down
A0A287AAR5	*NDUFAB1*	0.425	0.038	down
Q6UAQ8	*ETFB*	0.437	0.048	down
A0A076JEH2	*COX2*	0.466	0.046	down
F1RI18	*UQCRQ*	0.523	0.037	down
A0A4X1TIX1	*CAB39*	0.546	1.196×10^{-4}	down
A0A4X1VHS3	*ABCE1*	1.601	8.091×10^{-5}	up
A0A4X1T882	*MYOZ3*	2.028	3.733×10^{-4}	up
A0A287A8C2	*FLNC*	2.416	2.263×10^{-5}	up
A0A286ZSD6	*GPD2*	2.911	2.864×10^{-4}	up
A0A4X1VL95	*PPP1R14C*	4.826	1.832×10^{-4}	up

Note: (a) down indicates that this protein is highly expressed in Tibetan pigs, and up indicates that it is highly expressed in Duroc pigs.
